# Two-year clinical and radiographic evaluation of ACTIVA BioACTIVE versus Compomer (Dyract® eXtra) in the restoration of class-2 cavities of primary molars: a non-inferior split-mouth randomised clinical trial

**DOI:** 10.1186/s12903-024-04132-w

**Published:** 2024-04-10

**Authors:** Reda Banon, Jeroen Vandenbulcke, Jakob Van Acker, Luc Martens, Peter De Coster, Sivaprakash Rajasekharan

**Affiliations:** 1 Paediatric Dentistry, Oral Health Sciences, ELOHA (Equal Lifelong Oral Health for All) research group, Ghent University Hospital, Ghent, Belgium; 2 Department of Reconstructive Dentistry and Oral Biology, Oral Health Sciences, Ghent University Hospital, Ghent, Belgium

**Keywords:** Bioactive material, Bulk-fill composite, Primary molars

## Abstract

**Objectives:**

The trial aimed to compare the clinical performance and radiographic success of ACTIVA BioACTIVE versus Compomer in restoring class-II cavities of primary molars.

**Materials and methods:**

A non-inferior split-mouth design was considered. A pre-calculated sample size of 96 molars (48 per group) with class-2 cavities of twenty-one children whose ages ranged from 5 to 10 years were randomly included in the trial. Pre-operative Plaque Index (PI), DMFT/dmft scores and the time required to fill the cavity were recorded. Over 24 months, the teeth were clinically evaluated every six months and radiographically every 12 months by two calibrated and blinded evaluators using the United States public health service (USPHS)-Ryge criteria. The two-sided 95% confidence interval (CI) for the difference in success rate was considered to assess non-inferiority, and the margin was set at -18%. The linear mixed model and Firth’s logistic regression model were used for data analysis (*P *< 0.05).

**Results:**

After 24 months, 86 teeth (43 per group) were evaluated. The mean PI score was 1.1(± 0.9), while DMFT/dmft was 0.35 (± 0.74) and 6.55 (± 2.25) respectively. The clinical and radiographic success rate of Dyract vs. ACTIVA was 95.3% and 88.3% vs. 93% and 86%, respectively. The two-sided 95% CI for the difference in success rate (-2.3%) was − 3.2 to 1.3% and didn’t reach the predetermined margin of -18% which had been anticipated as the non-inferiority margin. Clinically, ACTIVA had a significantly better colour match (*P* = 0.002) but worse marginal discolouration (*P* = 0.0143). There were no significant differences regarding other clinical or radiographic criteria (*P* > 0.05). ACTIVA took significantly less placement time than Dyract, with a mean difference of 2.37 (± 0.63) minutes (*P* < 0.001).

**Conclusion:**

The performance of ACTIVA was not inferior to Dyract and both materials had a comparable high clinical and radiographic performance in children with high-caries experience. ACTIVA had a significantly better colour match but more marginal discolouration. It took significantly less time to be placed in the oral cavity.

**Trial registration:**

The study was registered at ClinicalTrials.gov on 4 May 2018 (#NCT03516838).

## Introduction

Developing a suitable dental material is challenging as restorative dentistry becomes less invasive and more bioactive [1]. Difficulties can be encountered when treating uncooperative children or using restorative materials with different restorative clinical steps. These difficulties are even more pronounced in paediatric dentistry in children with high caries experience and uncooperative behaviour [2]. As a result, several restorative materials have been introduced in paediatric dentistry to overcome these issues [3].

Among them are compomers. They are widely used and evaluated in primary dentition, with proven high clinical success [4–18]. However, they do not retain any bioactive properties that might help to remineralise and bond to the remaining dentine structure. They require several pre-treatment steps and a multi-layering technique, which might be less attractive for some practitioners [19]. .

Resin-based composites (RBC) have been used due to their superior mechanical, aesthetic, and adhesion properties. However, shrinkage with subsequent microleakage remains a major problem, in addition to lack of the remaining tooth structure [20].

Glass ionomers cements (GIC) are also popular in paediatric dentistry for their simple application, fluoride release, and less moisture sensitivity [21]. On the other hand, they might have inferior mechanical properties, worse marginal adaptation, and a higher risk of restoration failure compared to RBC [22].

Bulk-fill resin composites that could be placed in a single increment of 4–5 mm have been developed to simplify the restoration procedure [23]. This procedure is also less susceptible to technical errors that arise from void incorporation between the composite increments, causing incomplete unity of the filling restoration [24]. Bulk-fill resin composites have shown similar clinical behaviour to conventional incremental RBC [25].

Another innovation is incorporating bioactive components to improve the tooth-filling bond, along with the traditional adhesion, through forming and integrating hydroxy apatite crystals within the dentinal tubules, thereby reducing the odds of marginal leakage and further secondary caries formation. In addition, the remineralisation potential of affected hard dental tissue may be beneficial in children with high caries experience [26].

ACTIVA BioACTIVE is an ionic composite resin that combines the biocompatibility, chemical bond, and the remineralising and fluoride-releasing ability of glass ionomer cement (GIC) with the mechanical properties, aesthetics, and durability of RBC. It can be placed in a single increment of 4–5 mm [27, 28]. In addition, it is claimed that this material has bioactive properties due to its bioactive fillers. However, this claim has no conclusive evidence to date [29, 30]. In vivo, ACTIVA had a similar performance up to one year follow-up period compared to bulk-fill composite and Giomer hybrid composite in primary dentition [31, 32], and to nanohybrid composite in permanent dentition [33].

Such a restorative material as ACTIVA might provide a reliable alternative treatment to the traditional restorative procedure with improved mechanical properties, bioactivity, ability to release fluoride, and the possibility of placement in 4–5 mm increments. However, up to date, there is scarce information in the literature about this restorative material, and there needs to be more reliable in vivo studies with long follow-up periods. Therefore, this study’s primary outcome is evaluating the clinical and radiographic performance of ACTIVA in class-II restorations in primary molars compared to a compomer (Dyract) in vivo based on non-inferiority assumption. The secondary outcomes are comparing the time needed for placement of ACTIVA in the cavity to Dyract and assessing the oral hygiene progression over time. The null hypothesis is that the clinical and radiographic performance of ACTIVA is inferior to Dyract.

## Materials and methods

### Study design

To compare ACTIVA and Dyract in vivo, a monocenter, non-inferiority prospective double-blinded (patient and evaluator) split-mouth randomised controlled trial (RCT) was conducted in Ghent University Hospital and reported according to CONsolidated Standards Of Reporting Trials (CONSORT) [34]. The type, chemical composition, and mode of cure of both materials are reported in Table 1. All methods were carried out in accordance with relevant guidelines and regulations, and the protocol was approved by the ethical committee of Ghent University Hospital (#B670201629533) and registered at ClinicalTrials.gov on 4 May 2018 (#NCT03516838). Parents signed the informed consent after receiving an oral explanation.

**Table 1:** Type, chemical composition, and mode of cure of all tested materials

Material	Type	Chemical composition	Mode of cure
Dyract® eXtra (Dentsply DeTrey GmbH) Konstanz, Germany	Poly acid modified composite	UDMA, Carboxylic acid modified dimethacrylate (TCB resin), (TEGDMA), Camphorquinone, Strontium-alumino-sodium-fluoro- silicate glass, and Strontium fluoride	Light-cure: an acid-base reaction occurs upon absorption of water from the surrounding
ACTIVA™ BioACTIVE (Pulpdent) MA, USA	Ionic composite resin	Diurethane dimethacrylate (UDMA) and other methacrylate-based monomers, polyacrylic acid/maleic acid copolymer, silanated bioactive glass and calcium, silanated silica, sodium fluoride, aluminoflurosilicate ionomer glass, and water	triple cure reaction; light-cure, self-cure resin, and self-cure glass ionomer reaction

### Sample size calculation

The sample size was calculated based on the reported success rate of Dyract in the literature, which ranged between 78 and 96%, with a mean of 87% [5–18]. This mean was in accordance with a meta-analysis [15].

At the time of sample size calculation, no in vivo information on ACTIVA was available in the literature. Therefore, a 6-months pilot study involving placement of 20 ACTIVA class-II restorations (other than the included restorations in the main trial) in primary molars was conducted, which resulted in a 95% clinical success rate.

Using Sealedenvelope™ Calculator (Sealed Envelope Ltd. 2012), and based on the assumption of binary outcome measures (success/failure) and non-inferiority trial while fixing the non-inferiority limit at 9%, as the success percentage of the control group ranged between 78 and 96% which was clinically acceptable, a total number of 70 teeth (35 teeth per group) was calculated to detect a significant difference for a two-sided type I error of 5% and a power of 80%. This number was increased to 39 teeth per group, allowing for a drop-out rate of 10%.

### Definition of non-inferiority margin

The non-inferiority margin was based on the success rate of Dyract in studies which are reported in the previous section, which covered a range of 18% (78–96%). Since this variation was showed by the standard treatment, the performance of ACTIVA was considered non-inferior if the success rate, including 95% confidence interval (CI), falls within this variation. Therefore, for the comparison between Dyract and ACTIVA, Δ = -18% was defined as the non-inferiority margin.

### Randomisation and blinding

Recruited teeth were randomly assigned following simple procedure to either of the two groups (ACTIVA or Dyract) based on pre-generated sequence. The sequence was generated by an independent person using Random Integer Generator (RANDOM.ORG, Randomness and Integrity Services Ltd.). These sequences were kept in sequentially numbered, opaque sealed envelopes and the operator was unaware. The allocation ratio was set to be equal between both treatment groups (1:1) by recruiting an equal number of carious teeth on each side of each subject’s mouth. The required teeth were not always matched and could be the first or second molar from both the upper and lower jaw to recruit as many teeth as possible. In each visit, a quadrant is prepared for material placement while the operator was blinded until the material placement. Then, a second person opened the sealed envelope, revealed the type of restoration, and assigned participant to intervention, as both materials have a distinguished application method. In the second visit, the other side is prepared, and the second person disclosed the treatment. Both participants and evaluators were also blinded to intervention by replacing the type of treatment in the patient’s dossier by code which is only known by the independent person who conducted the randomisation. When the trial is completed and all teeth were evaluated, the independent person replaced the codes with the type of treatment and matched the groups.

### Patient selection and eligibility criteria

#### Patient inclusion criteria

Only healthy cooperative children with the score I American Society of Anaesthesiologists (ASA), from both genders, aged between five to ten years with at least one carious vital primary molar on each side (split-mouth) were recruited.

### Tooth inclusion criteria

Clinically, recruited teeth were first and second primary molars from both sides and both jaws (upper and lower) with proximal dentin caries with an International Caries Detection and Assessment System (ICDAS) score of 4 (underlying dark shadow from dentin) or 5 (Distinct cavity with visible dentine) [35]. Radiographically, the proximal caries were confined to the outer half of the dentin, with a predicted survival of at least two years until normal exfoliation.

All included teeth were vital, restorable, and free of any symptoms. Uncooperative children, mutilated teeth, teeth with extensive caries, formative dental defects, pathological mobility, pulp exposure, or indication for pulp therapy were excluded.

**Fig. 1:**
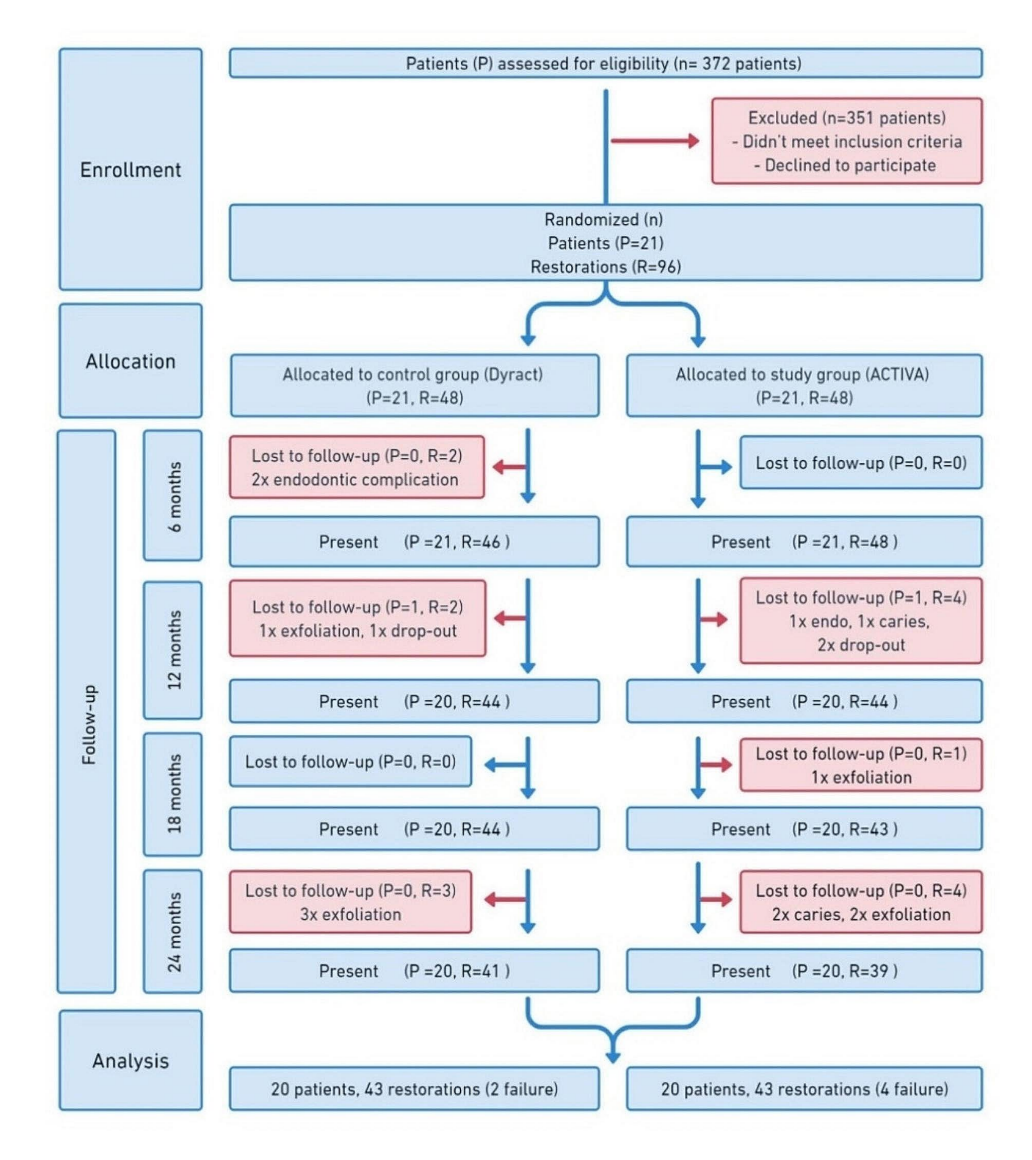
Flowchart illustrating patient enrolment, allocation, follow-up, and analysis. n = number, P = patient, R = restoration

At baseline, the mean pre-operative plaque index (PI) by Silness and Löe 1964 [36] and caries index (CI) DMFT & dmft scores by Klein, Palmer and Knutson 1938 for permanent and primary teeth [37] were recorded. The operator recorded the mean PI once again in the second follow-up visit (12 months) to investigate the progression of oral hygiene status.

### Clinical procedure

One master student treated all children to avoid inter-operator bias. A pre-operative bitewing radiograph was taken for diagnosis (as part of the routine dental check-up). The tooth was anesthetised using local anaesthesia (Septanest Normal 4% articaine with 1/200,000 adrenaline, Saint-Maur-des-Fossés, France) and isolated using a rubber dam (Isodam, Sigma dental, Derbyshire, UK). Class II restorations were prepared using a high-speed diamond pear bur (ISO 806 314 234 524) with ample water spray. A low-speed carbide round bur (H1.204.014) or hand excavator was used to remove further deeper caries.

Subsequently, a metal matrix band (V3 Sectional Matrix System™, Dentsply DeTrey, Konstanz, Germany) and a wooden wedge were placed interdentally. At this point, the tooth was assigned to the allocated group according to randomisation, and both restorative materials were placed according to the manufacturer’s instructions.

The cavity was etched with 35% phosphoric acid (Ultra-Etch®, Ultradent, South Jordan, UT, USA) for 20 s and then washed and dried. Next, a bonding agent (Prime & Bond NT, Dentsply DeTrey, Konstanz, Germany) was applied and cured for 10 s. Dyract was placed in layers up to 2 mm, while ACTIVA was placed in bulk up to 4 mm.

Both materials were cured using a light-cure source (Satelec Mini LED curing light, A-dec Inc., Newberg, USA) with a light intensity of 1250 mW/cm^2^ and wavelength 420–480 nm for 20 s. Finally, the material was finished using a diamond finishing bur (ISO198/018 TR-13EF) and polished using a silicon rubber polishing bur, and the occlusion was checked for any interferences using articulating paper.

### Follow-up, evaluation, and calibration

Over 24 months, the teeth were evaluated clinically every six months using the United States Public health Service (USPHS) Ryge criteria [38] and radiographically (presence or absence of peri-radicular radiolucency and secondary caries) every twelve months by two calibrated and blinded paediatric dentists (V.J, V.A.J). Inter- and intra-evaluator calibration was based on observing 20 clinical pictures and ten class-II restored Frasaco teeth with different clinical situations.

Based on other studies in the literature in determining the success and failure rate, both Alpha (A) and Bravo (B) scores were considered a clinical success, and the score Charlie (C) was recorded as a failure. Other conditions, such as normal exfoliation, caries in another tooth surface, and severe gingival inflammation, were not considered a failure because they are not influenced by the treatment.

### The time needed for placement

The duration of the restoration procedure was recorded in minutes using a timer by a person other than the operator in order to investigate whether one material takes more time than the other to be placed in the oral cavity. The starting point was after placing the matrix band and interdental wedge, and ended when the restoration finishing was completed.

### Statistical analysis

Since the design was split-mouth, some variables were equal among the treatment groups (i.e. age, gender, baseline PI, DMFT and dmft). Therefore, only descriptive statistics were performed to report those variables.

Inter- and intra-examiner agreement was calculated using Cohen’s Kappa statistical test, and the values were interpreted using Landis & Koch scores 1977 (Table 2) [39].

**Table 2:** Scores interpretation according to Landis & Koch [1]

Score	0	0–0.2	0.21–0.4	0.41–0.6	0.61–0.8	0.81–1
Value	Poor	Slight	Fair	Moderate	Substantial	Almost prefect

A two-sided 95% CI for the difference in success rate was used to assess non-inferiority. A generalised linear mixed model and Firth’s logistic regression model were used to evaluate the clinical and radiographic outcomes. The material, follow-up visit, and jaw were considered the main variables in all models. In addition, the interaction between “material” and “visit” was tested for significance to determine whether the evolution of the responses over time (visits) is different for the two materials.

Before building each model, it was decided whether to consider an intercept and a slope for each patient. This was done by considering two intercept-only models: one with random intercepts and one with both random intercepts and slopes. Both models were then compared, and when no difference in the Akaike Information Criterion (AIC) was found, the simpler model with random intercepts was chosen. If the difference in AIC between the two models was greater than 4, the models were considered different, and the model with the smaller AIC was accepted to provide a better fit. The “bglmer” function from the blme package was used, allowing a penalized maximum likelihood estimation method to account for the low frequencies present for the response categories of interest.

The repeated measures of PI at baseline and 12 months follow-up were assessed using the regression model fit. ANOVA was used to compare placement time between the materials.

R software was used to perform the statistical analysis of the in vivo data. Graphs and figures were designed using GraphPad Prism 9. The significance level was set at (*P* < 0.05).

## Results

Subjects were recruited over two years period. The trial was ceased when the sample size was reached. After 24 months evaluation and out of the 48 randomly assigned teeth (21 children), seven teeth were lost due to exfoliation, and one child (four teeth including one exfoliated tooth) withdrew from the study on own will; thus data from 20 children (43 teeth in each group) were available for analysis. The patient selection flowchart and descriptive statistics of patients included in the analysis are shown in Fig. 1; Table 3. The result of Cohen’s kappa (κ) for inter- and intra- evaluator agreement was 0.75 (substantial) and 0.81 (almost perfect), respectively (according to Landis & Koch’s interpretation).

**Table 3:** Descriptive statistics of children included in the analysis, SD = standard deviation

	Value								Total
Age (years)	Range				Mean (SD)				-
	5–10				7.3 (1.49)				
Per gender	Male				Female				20
	5				15				
Per subject treated	Children				Teeth				-
	20				86				
Per material	Dyract				ACTIVA				86
	43				43				
Per jaw	Maxillary				Mandibular				86
	48				38				
Per molar	First molar				Second molar				86
	44				42				
Per tooth	54	55	64	65	74	75	84	85	86
	11	13	11	13	9	7	13	9	
Mean PI	Baseline 1.1 (0.9)				12 months follow-up 0.6 (0.275)				*P* < 0.01
DMFT/dmft	DMFT 0.35 (0.74)				dmft 6.55 (2.25)				-

The clinical and radiographic success rate of Dyract was 95.3% and 88.3%, while for ACTIVA, it was 93% and 86%, respectively. The two-sided 95% CI for the difference in success rate (-2.3%) was − 3.2 to 1.3%, and didn’t reach the predetermined non-inferiority margin of -18%, which was required for the non-inferiority of the treatment group.

**Fig. 2:**
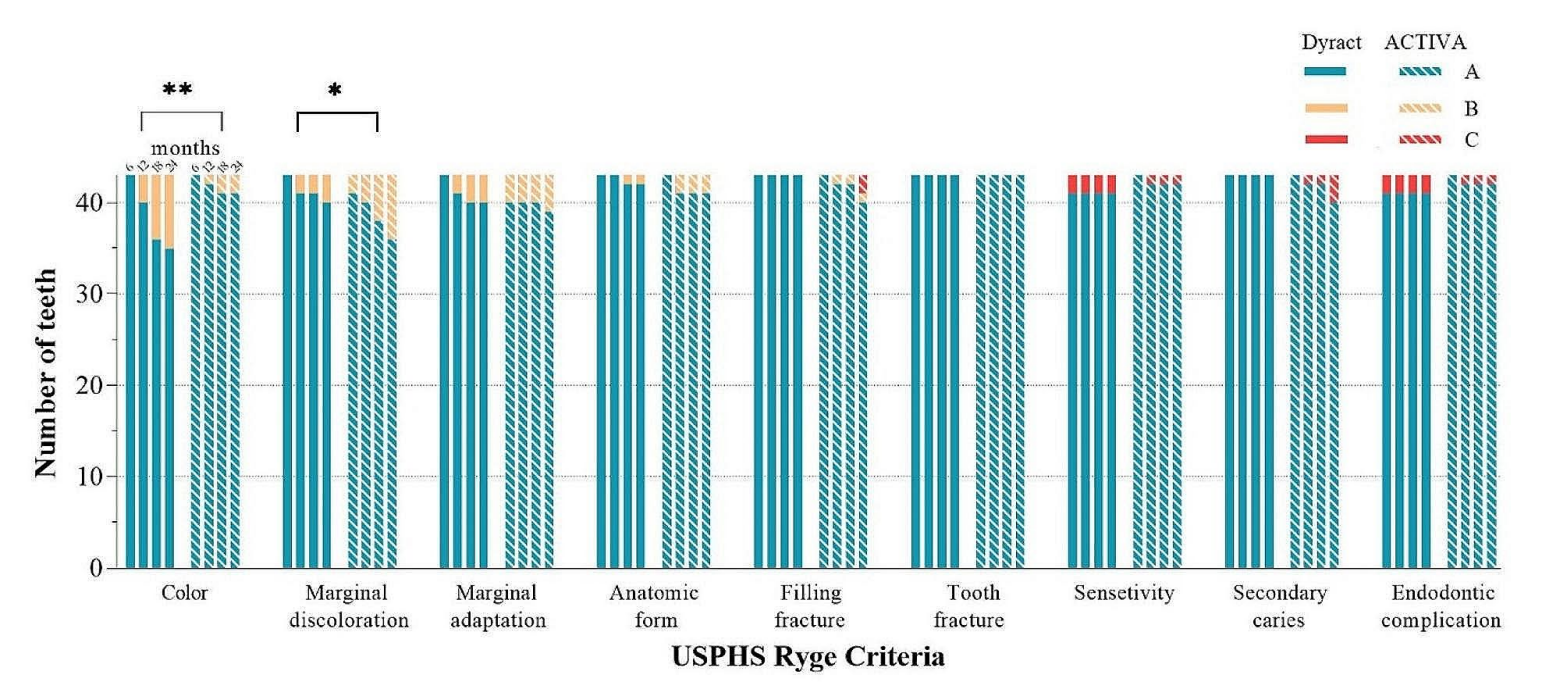
Clinical evaluation based on USPHS Ryge Criteria at 6, 12, 18, and 24 months. Score A represents the ideal clinical situation, score B represents a clinically acceptable situation, and score C represents failure. Both groups had a significant difference regarding colour match (*P* = 0.002) and marginal discolouration (*P* = 0.0143)

The clinical evaluation based on USPHS Ryge Criteria of both groups over 24 months is illustrated in Fig. 2. Compared to Dyract, ACTIVA had a significantly better colour match (*P* = 0.002) but more marginal discolouration (*P* = 0.0143). There was no statistically significant difference regarding marginal adaptation (*P* = *0.138*), anatomic form (*P* = *0.269*), gross restoration fracture (*P* = *0.156*), tooth fracture, sensitivity (*P* = *0.497*), secondary caries (*P* = *0.395*), and endodontic complications (*P* = *0.497*). Two teeth restored with Dyract and one restored with ACTIVA had an endodontic complication in the first year without secondary caries.

**Fig. 3:**
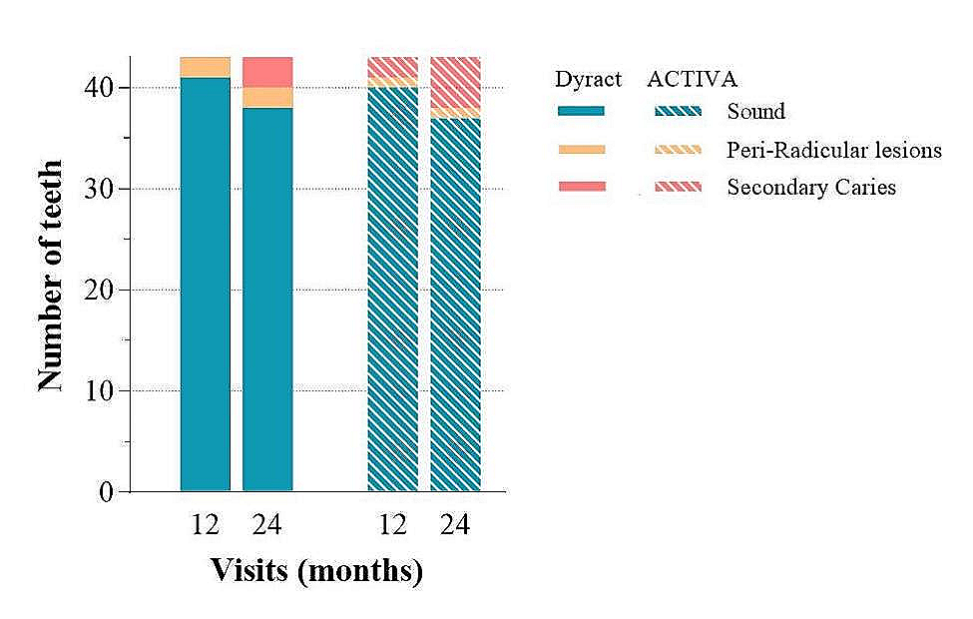
Radiographic evaluation at 12 and 24 months. No significant difference was detected (*P* > 0.05)

Radiographically, two teeth from the Dyract group developed a peri-radicular radiolucency and three secondary caries. While in the ACTIVA group, one tooth showed a peri-radicular radiolucency and four secondary caries (Fig. 3). The estimated probability of having radiographic complications was the same for both materials across the follow-up time points (*P* = *0.79*).

**Fig. 4:**
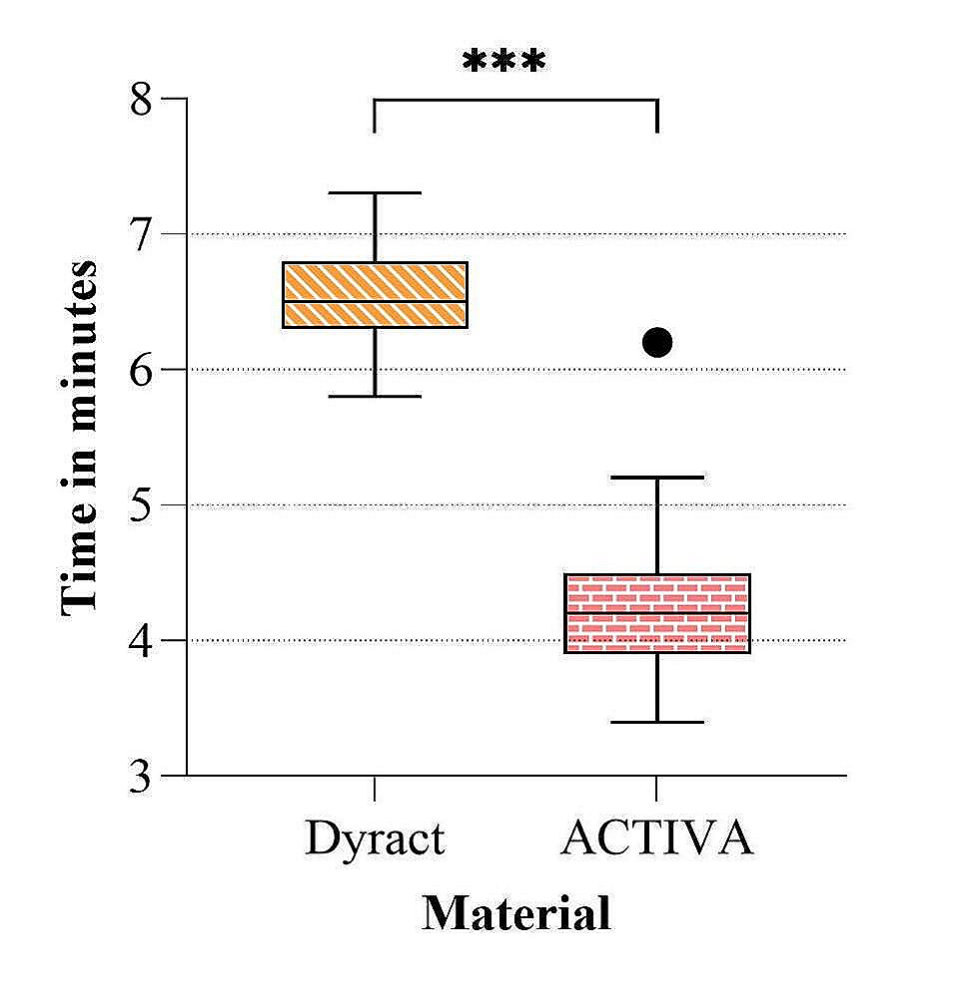
Time needed to place each material in the oral cavity during the restoration procedure. A significant difference was found between both materials (*P* < 0.001)

The baseline assessment of oral hygiene status showed a median (IQ) plaque index PI of 1.1 (0.9), which was considered fair. However, this record of PI decreased to 0.6 (0.275) at 12 months follow-up (*P* < 0.01). Based on the fitted model (Fig. 4), ACTIVA took significantly less time than Dyract to be placed in the oral cavity during the restoration procedure, with an estimated difference of 2.37 (± 0.63) minutes (*P* < 0.001). In addition, the variability in placement time appeared to be similar across the materials. There appeared to be an outlying placement time for one patient in the ACTIVA group.

## Discussion

Dyract was chosen as the control group because it is widely used, and its performance in primary dentition is well documented. A split-mouth design was adopted because it allows each subject to act as its control and requires less sample size for the same power [40]. In addition, no cross-contamination that might lead to false positive results is expected, as the treatment effect is confined to the treated tooth [41]. The recruited teeth from both sides of the mouth were matched as much as possible, meaning both first and second molars from the upper and lower jaws were included. Tooth anatomy, size, and position in the mouth might affect the restoration performance but would allow including more teeth. Nevertheless, no statistical significance was found in the restoration performance or placement time between the first and second molar, and between the upper and lower jaw. The calculated sample size of 35 teeth per group was based on the success rate of Dyract from the literature, which ranged between 14 and 104 teeth with a mean of 50 teeth per group [5–18].

The population represented by the included subjects comprised mainly children with a high caries experience and a fair baseline oral hygiene. These findings indicate that both materials would perform equally or better in a normal population with better oral hygiene status. In addition, the PI scores had improved at the 1-year follow-up visit, most probably due to the repeated oral hygiene instructions and follow-up.

The clinical and radiographic success rates of ACTIVA were not inferior to Dyract and within the non-inferiority margin. Therefore, the null hypothesis was rejected. The clinical success rate of Dyract was consistent with other studies. Hse and Wei reported a 95% clinical success over 12 months [6], Gross et al. reported 96% over 24 months [12], while Ertugul et al. reported 95.7% over the same period [16]. Other studies have reported success rates ranging between 78% and 96% (mean 87%) [5–18].

ACTIVA was evaluated in vivo for a 12-month follow-up period. Bhadra et al. reported a failure of 2 out of 28 analysed teeth, with a clinical performance comparable to nanohybrid composite in permanent dentition [33]. Deepika et al. reported no failure in the primary dentition, only a change in the colour and marginal discolouration, which was consistent with our study. However, the clinical performance was better compared to Giomer [32]. Lardani et al. reported 2% failure with a performance comparable to SDR for both functional and aesthetic properties in the primary dentition [31].

The wide variation in success rates in literature could be due to differences in the follow-up period (one to three years), the type of the study (split-mouth, RCT or non-RCT) where different statistics were used, operator experience, the use of rubber dam, and evaluator’s reliability [18, 42]. Patient’s age and cooperation are other factors contributing to outcome differences. An 11-months median survival time of restorations placed in 3-year-old children was reported. This value increased to 44 months when studied in children aged 7 to 8 years [43]. Only cooperative children aged between 5 and 10 years were included in our study.

Over the 24 months, Dyract went through significantly more colour changes than ACTIVA, which is probably related to abrasive surface wear and pH fluctuations. When Dyract was abraded with a toothbrush, it suffered the highest mass loss and the most significant reduction in surface hardness compared to a flowable and hybrid resin composite [44]. When Dyract was exposed to Coca-Cola and orange juice, unfavourable colour changes of more than ΔE = 11.0 were seen compared to composite resin. This can be explained by the low pH that softens the surface of the compomer, resulting in the separation of structural ions from the glass phase. Subsequently, individual particles dissociate, leaving a rough surface with voids [45]. When ACTIVA was immersed in different consumable solutions, it maintained a clinically acceptable colour (ΔE 3.0–8.0) which was better than Filtek nanocomposite [46], Giomer and Fuji II [47] but worse than Filtek nanohybrid composite resin [48], which indicates that ACTIVA is more composite than RM-GIC.

On the other hand, ACTIVA exhibited a more pronounced marginal discolouration than Dyract. This could be due to the difference in the coefficient of thermal expansion between the restoration and the tooth structure. Due to the force of mastication and the intake of hot and cold consumables, a negative interfacial pressure is generated, forcing the oral fluid to infiltrate into the margins and causing some degree of discolouration [49]. This was observed when ACTIVA was compared to Filtek™ Supreme Ultra resin composite and showed more marginal discolouration [50]. This phenomenon results in an increased degree of microleakage, which might explain the failed teeth in the ACTIVA group due to secondary caries.

Furthermore, when the bonding step was omitted, ACTIVA showed a worse marginal adaptation than Ceram X Mono in vitro [51] and an annual failure rate of 24.1% after one year in vivo [52]. Therefore, placing ACTIVA without pre-treating the cavity with etch-and-bond systems is highly discouraged. Nevertheless, in this study, all cavities were pre-treated, and the difference in marginal adaptation between both materials was not significant. No clinical secondary caries was found in the Dyract group at any point, although the reported main reason for failure for compomer in the primary dentition is secondary caries [18].

ACTIVA significantly took 2.37 ± 0.63 min less than Dyract to be placed in the prepared cavities as it can be placed in bulk up to 4 mm increments. This is due to the monomer composition and the chemical cure resin in ACTIVA, which allows the material to set fully even in the deeper layers [53]. In contrast, the light cure is essential to initiate the setting reaction of Dyract [54]. In addition, Dyract has to be condensed and adjusted to mould the anatomy. ACTIVA, which is flowable, requires less handling, resulting in a shorter working time. Considering that the cavities in the current study were all class II, no cusp build-up or extensive restorations were performed.

The application time difference of 2.37 min between the two materials may have had only a limited impact compared to the total period for the dental visit. Still, those two minutes are tangled in the most critical part of the dental visit. The cavity should stay dry, while the child usually has been on the chair for a while and should stay still and keep his mouth open. Therefore, reducing the treatment time by two minutes could be interesting to shorten the most technique-sensitive step in the procedure.

One of the limitations of this study is that the operator was only blinded until material placement, as both interventions have different application procedures. The sample size was considerably small compared to other studies, and the in vivo efficiency of the bioactive properties of ACTIVA could not be demonstrated in the final results, probably due to the short follow-up period. Therefore, clinical studies with larger sample sizes and extended follow-up periods are essential to assess the ability of a bioactive restorative material to prevent secondary caries or stop the active progression.

## Conclusion

The performance of ACTIVA was not inferior to Dyract and both materials had a comparable high 24-month clinical and radiographic performance in children with high-caries experience. ACTIVA took significantly less time than Dyract to be placed in the oral cavity, which could be of interest for the dentist to reduce the chairside time.

## Data Availability

The datasets generated and/or analysed during the current study are not publicly available due to Patients’ confidentiality but are available from the corresponding author on reasonable request after de-identification.
